# Facile Creation of 3-Substituted-3-Hydroxy-2-Oxindoles by Arginine-Catalyzed Aldol Reactions of α,β-Unsaturated Ketones with Isatins

**DOI:** 10.3390/molecules181214505

**Published:** 2013-11-25

**Authors:** Tingting Yan, Xiaoyan Wang, Hongbao Sun, Jie Liu, Yongmei Xie

**Affiliations:** 1 State Key Laboratory of Biotherapy, West China Hospital, West China Medical School, Sichuan University, Chengdu 610041, China; E-Mails: yttscu@163.com (T.Y.); hongbaosun@163.com (H.S.); 2 Analytical & Testing Center, Sichuan University, Chengdu 610064, China; E-Mail: wangxiaoyan@scu.edu.cn

**Keywords:** 3-substituted-3-hydroxy-2-oxindoles, arginine, aldol reaction, isatins, α,β-unsaturated ketones

## Abstract

An efficient approach for the synthesis of 3-substituted-3-hydroxy-2-oxindoles has been achieved via an aldol reaction of α,β-unsaturated ketones and isatins using arginine as an organocatalyst. A range of 3-substituted-3-hydroxy-2-oxindoles were obtained in moderate to high (up to 99%) yields. These 3-substituted-3-hydroxy-2-oxindoles with an additional enone moiety provide an opportunity for further elaboration of the products and for potentially interesting biological activities. In addition, the formation of 3-substituted-3-hydroxy-2-oxindole **3a** was conﬁrmed by X-ray crystallography. The possible reaction mechanism reveals that the reaction proceeds via a double action process.

## 1. Introduction

**Figure 1:**
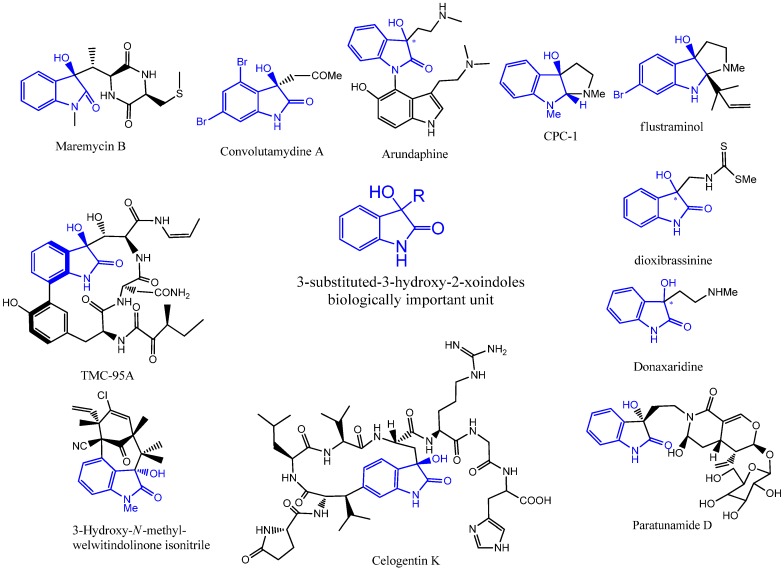
Biologically important molecules containing 3-substituted-3-hydroxy-2-oxindoles.

3-Substituted-3-hydroxy-2-oxindoles are heterocyclic organic compounds that possess a carbonyl group at the 2-position of the 5-membered ring and a quaternary carbon centre at the 3-position of this ring. This kind of compounds have become important synthetic targets as these structural frameworks form the core units of many natural products and pharmaceutically active compounds [1]. Convolutamydines [2], arundaphine [3], donaxaridine [4], dioxibrassinine [5], maremycins [6], paratunamide [7], celogentin K [8], TMC-95A-D [9], flustraminol [10], 3-hydroxy welwitindolinones [11] and CPC-1 [12] are some examples of a growing list of bioactive 3-substituted-3-hydroxy-2-oxindole natural products (Figure 1).

**Scheme 1:**
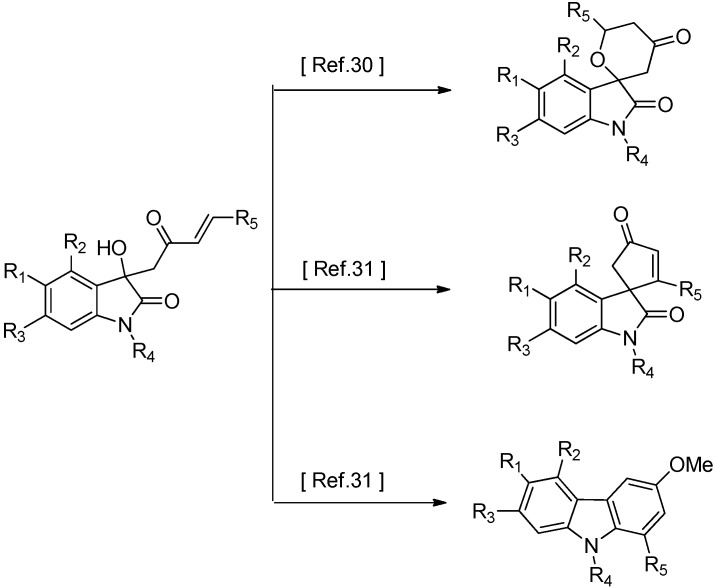
Further elaboration of 3-hydroxy-3-(2-oxo-4-arylbut-3-enyl) indolin-2-one products.

They display diverse biological and pharmacological activities such as potent antioxidant, anticancer, anti-HIV, and neuroprotective properties. Owing to the signiﬁcance of this structural motif, numerous elegant synthetic methodologies have been developed [13,14,15,16,17,18,19,20,21] and aim to facilitate the synthesis of sufficient quantities of the desired natural products and related analogues for biological evaluation and structure-activity relationship studies, and thus finally contribute to the development of new therapeutic agents or important biological tools. The most direct approach to 3-substituted-3-hydroxy oxindoles is a nucleophilic addition of appropriate nucleophiles to isatins, such as the aldol reaction or an alkylation of isatins. Recently, several elegant approaches to 3-aryl or alkyl-3-hydroxyindolin-2-ones via the cross-aldol reaction between isatins and ketones have been extensively studied [22,23,24,25,26,27,28,29,30,31]. However, few examples of the corresponding aldol reactions of various α,β-unsaturated ketones with isatins were found in the literature. The aldol addition of an α,β-unsaturated ketone to isatin could produce 3-substituted-3-hydroxy-2-oxindoles with an additional enone moiety and thus provide a chance for further elaboration of the products (Scheme 1) and perhaps different or improved biological activities [30,31]. The formation of quaternary carbon centers and the chemoselectivity (α,β-unsaturated ketone as a nucleophile is arising from the inherent multiple reactivity that involve ketone, β-carbon and methylene unit) via addition of α,β-unsaturated ketones to isatins represents a major challenge and has stimulated many a synthetic chemist.

**Scheme 2:**
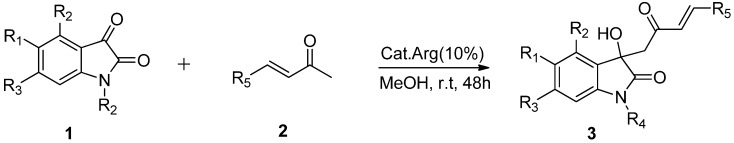
Synthesis of 3-substituted-3-hydroxy-2-oxindoles.

α-Amino acids are readily available organic molecules, which have so far been utilized as chiral auxiliaries, chiral ligands and chiral synthons for natural products and drugs. Using amino acids as a catalyst over other organic molecules could provide considerable green technology beneﬁts because amino acids are a component of natural proteins and completely biodegradable in nature [32,33,34,35]. Since List, Lerner, and Barbas first reported the proline-catalyzed direct aldol reaction [36], other amino-acid based organocatalysts have been developed as mimics of enzymes for various reactions. The mechanism in the proline-mediated reactions is based on the initial formation of an imine between the nitrogen atom of proline and the carbonyl group of the substrate and then conversion to an enamine [37]. Such an imine can be formed by other amino acids and thus the other amino acids could catalyze similar reactions. In view of this mechanism, we wish to further extend the use of amino acids as organocatalysts. Herein, we describe the use of amino acids for the facile synthesis of 3-substituted-3-hydroxy-2-oxindole frameworks in high yields (up to 99%) via the aldol reactions of α,β-unsaturated ketones and isatins (Scheme 2).

## 2. Results and Discussion

**Figure 2:**
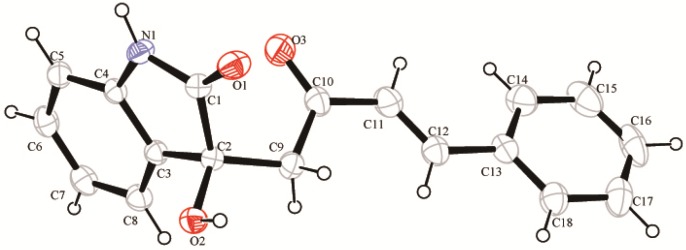
X-ray crystal structure of **3a**.

In our initial studies, the direct aldol reaction between isatin (**1a**) as an acceptor and (*E*)-4-phenylbut-3-en-2-one (**2a**) as a donor with a catalyst loading of 20 mol% in MeOH at room temperature was selected as a benchmark for catalyst evaluation. Some screening results are listed in Table 1. The simple amino acids bearing only an amino group and a carboxyl group could not catalyze this reaction. No expected aldol adducts were observed after direct determination by TLC (Table 1, entries 1–5). This indicated that it is impossible for the aldol reaction in which these primary amine and secondary amine catalysts unilaterally activate the (*E*)-4-phenylbut-3-en-2-one (**2a**) to form an enamine intermediate. Then, we turned our attention to other complex amino acids such as arginine, tryptophan and histidine as catalysts (Table 1, entries 6–8). Pleasingly, arginine, an amino acid skeleton with a guanidine group, was effective for the reaction, affording the desired product **3a** in 88% yield (Table 1, entry 7). The formation of **3a** was conﬁrmed by X-ray crystallography (Figure 2) [38]. Although l-arginine was used as a catalyst, the product was obtained as a racemic mixture. The decrease of the reaction temperature down to 0 °C did not alter the enantioselectivity (data not shown).

**Table 1:** Catalyst screening and reaction conditions optimization ^a^.

Entry	Catalyst	Solvent	Time/h	Yield (%) ^b^
1	Proline	MeOH	24	N.D ^c^
2	Phenylalanine	MeOH	24	N.D
3	Leucine	MeOH	24	N.D
4	Tyrosine	MeOH	24	N.D
5	Tyrosine methyl ester	MeOH	24	N.D
6	Histidine	MeOH	24	N.D
7	Arginine	MeOH	24	88
8	Tryptophan	MeOH	24	N.D
9	Boc-Arginine	MeOH	24	N.D
10 ^d^	Arginine	MeOH	24	79
11 ^e^	Arginine	MeOH	24	91
12 ^f^	Arginine	MeOH	24	76
13 ^g^	Arginine	MeOH	24	44
14 ^e,h^	Arginine	MeOH	12	49
15 ^e,h^	Arginine	MeOH	24	52
16 ^e^	Arginine	MeOH	12	55
17 ^e^	Arginine	MeOH	48	98

^a^ Unless indicated otherwise, the reaction was carried out in 0.1 mmol scale in solvent (1.0 mL) at 25 °C for 24 h, and the ratio of **1a**/**2a**/catalyst is 1:1:0.2; ^b^ Isolated yield based on isatin; ^c^ N.D. refers to no reaction; ^d^ Catalyst loading is 5 mol%; ^e^ Catalyst loading is 10 mol%; ^f^ Catalyst loading is 30 mol%; ^g^ Catalyst loading is 50 mol%; ^h^ Temperature is 60 °C.

To further improve the yields, efforts were made to optimize other reaction parameters including solvents, catalyst loading and reaction temperatures. Thus, the reaction was studied in different solvents that included CH_2_Cl_2_, THF, dioxane, CH_3_CN, toluene, EtOH, *i*-BuOH, *n*-BuOH and H_2_O, but no better result was obtained. In general, reactions carried out in protic solvents gave better yields than those in aprotic solvents. This may be caused by poor solubility of arginine in aprotic solvents and H_2_O. Catalyst loading inﬂuenced the rate of the reaction. As the catalyst loading increased to 10 mol %, the yield increased steadily (Table 1, entries 7, 10, 11). Further improvement, however, was not achieved by further increasing the catalyst loading (Table 1, entries 12, 13). Temperature also inﬂuenced the rate of the reaction. Elevating the reaction temperature resulted in a low yield, while conducting the reaction at 60 °C gave a certain amount of byproducts (Table 1, entries 14, 15). The relatively higher yield of reaction could be compensated by prolonging reaction time to 48 h, and up to 98% yield was obtained (Table 1, entry 17). Through extensive screening, the optimized catalytic system was found to be **1a**/**2a**/arginine = 1/1/0.1, 1.0 mL MeOH as solvent at 25 °C for 48 h.

Having the optimized conditions in hand, the aldol reaction of α,β-unsaturated ketones with different structures was investigated. As shown in Table 2, a variety of α,β-unsaturated ketones proved to be excellent nucleophiles for this reaction, and provided the corresponding 3-substituted-3-hydroxy-2-oxindoles in good yields (up to 98%) (Table 2, entries 1–15). The electronic properties and steric hindrance of the substituents at the aromatic ring inﬂuenced the yields slightly. Generally, α,β-unsaturated ketones with electron-donating groups gave higher yields than those with electron-withdrawing groups (Table 2, entries 2, 10 *vs.* 3–8). *Ortho*- and *meta*-substituted α,β-unsaturated ketones gave higher yields than *para*-substituted α,β-unsaturated ketones (Table 2, entries 2‒5 *vs.* 8‒11). α,β-Unsaturated ketones bearing naphthyl and heterocyclic substituents participated in smooth aldol reactions in 81% and 88% yield, respectively (Table 2, entries 13, 14). Moreover, it is worthwhile to note that an α,β-unsaturated ketones derived from an aliphatic aldehyde was investigated and it was transformed with moderate yield (Table 2, entry 15). To further extend the application of arginine, substituted isatins with several representative α,β-unsaturated ketones were also examined (Table 2, entries 16–18). Incorporating protecting groups on the N1 of oxindole had no effect on reactivity, and gave the desired product in almost quantitative yield (99%) (Table 2, entry 16). However, the electronic properties of the substituents at the isatin affected the yields strongly (Table 2, entries 17–20). Isatin with an electron-donating group only gave 60% yield.

**Table 2:** Substrate scope for the aldol reaction of isatins and α,β-unsaturated ketones ^a^. 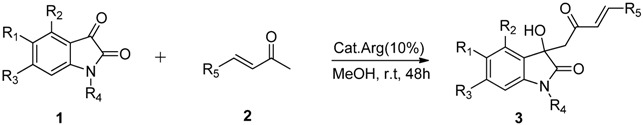

Entry	1	2	3	Yield (%) ^b^
1	R_1_ = R_2_ = R_3_ = R_4_ = H	R_5_ = Ph	**3a**	98
2	R_1_ = R_2_ = R_3_ = R_4_ = H	R_5_ = 4-MeO-C_6_H_4_	**3b**	95
3	R_1_ = R_2_ = R_3_ = R_4_ = H	R_5_ = 4-F-C_6_H_4_	**3c**	87
4	R_1_ = R_2_ = R_3_ = R_4_ = H	R_5_ = 4-Cl-C_6_H_4_	**3d**	80
5	R_1_ = R_2_ = R_3_ = R_4_ = H	R_5_ = 4-Br-C_6_H_4_	**3e**	82
6	R_1_ = R_2_ = R_3_ = R_4_ = H	R_5_ = 4-NO_2_-C_6_H_4_	**3f**	75
7	R_1_ = R_2_ = R_3_ = R_4_ = H	R_5_ = 3-CF_3_-C_6_H_4_	**3g**	86
8	R_1_ = R_2_ = R_3_ = R_4_ = H	R_5_ = 3-Br-C_6_H_4_	**3h**	85
9	R_1_ = R_2_ = R_3_ = R_4_ = H	R_5_ = 3-Cl-C_6_H_4_	**3i**	82
10	R_1_ = R_2_ = R_3_ = R_4_ = H	R_5_ = 3-MeO-C_6_H_4_	**3j**	96
11	R_1_ = R_2_ = R_3_ = R_4_ = H	R_5_ = 2-F-C_6_H_4_	**3k**	92
12	R_1_ = R_2_ = R_3_ = R_4_ = H	R_5_ = 3,4-diCl-C_6_H_4_	**3l**	73
13	R_1_ = R_2_ = R_3_ = R_4_ = H	R_5 _= 2-Naphthyl	**3m**	81
14	R_1_ = R_2_ = R_3_ = R_4_ = H	R_5_ = 2-Furyl	**3n**	88
15	R_1_ = R_2_ = R_3_ = R_4_ = H	R_5_ = Propyl	**3o**	67
16	R_1_ = R_2_ = R_3_ = H, R_4_ = CH_2_Ph	R_5_ = Ph	**3p**	99
17	R_1_ = Me, R_2_ = R_3_ = R_4_ = H	R_5_ = Ph	**3q**	60
18	R_1_ = Br, R_2_ = R_3_ = R_4_ = H	R_5_ = Ph	**3r**	99
19	R_3_ = Cl, R_1_ = R_2_ = R_4_ = H	R_5_ = Ph	**3s**	93
20	R_2_ = Cl, R_1_ = R_3_ = R_4_ = H	R_5_ = Ph	**3t**	92

^a^ The reaction was carried out in 0.1 mmol scale in MeOH (1.0 mL) at 25 °C for 48 h, and the ratio of **1**/**2**/arginine is 1:1:0.1; ^b^ Isolated yield based on isatins.

**Scheme 3:**
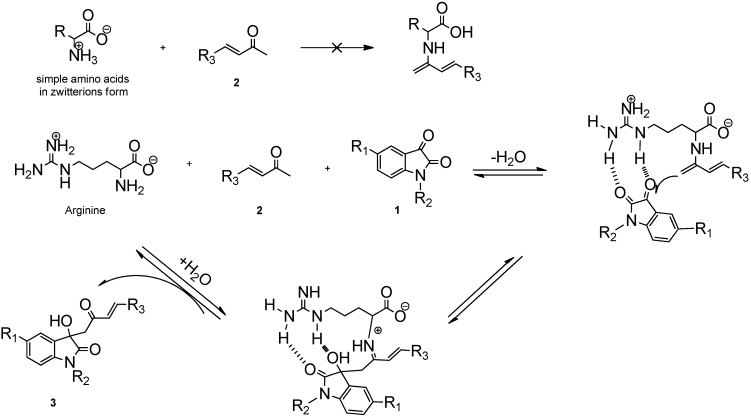
The double action catalysis mechanism.

Based on the results (Table 1, entries 1–9) and previous reports, [39,40,41] a possible reaction mechanism for the formation of 3-substituted-3-hydroxy-2-oxindoles could be proposed. As depicted in Scheme 3, in solid form or in polar solutions, amino acids can exist as zwitterions, which contain a protonated amino group and deprotonated carboxylate group. It is obvious that simple amino acids (only bearing an amine group and a carboxyl group) in zwitterionic form could not be considered as a base and therefore did not participate in this reaction (Table 1, entries 1–5). However, arginine, as a basic amino acid which possesses side chain basicity (pKa = 12.48) due to its guanidine group, can take a proton from the protonated amino group thereby resulting in the formation of the naked amino group.

Then, the naked amino group can also form an enamine with the carbonyl group of the substrate (Table 1, entries 7, 9) and the guanidine group presumably activates the carbonyl group of isatin (Table 1, entries 6–8). This double action of the amino acid may play a key role in this reaction and hence facilitate the aldol reaction.

## 3. Experimental

### 3.1. General

All chemicals were obtained from commercial sources and used without further purification. Column chromatography was carried out on silica gel (300–400 mesh, Qingdao Marine Chemical Ltd., Qingdao, China). Thin layer chromatography (TLC) was performed on TLC silica gel 60 F254 plates. ^1^H-NMR spectra were recorded on a Bruker AVII-400 MHz NMR spectrometer. The chemical shifts were recorded in ppm relative to tetramethylsilane and with the solvent resonance as the internal standard. Data were reported as follows: chemical shift, multiplicity (s = singlet, d = doublet, t = triplet, q = quartlet, m = multiplet), coupling constants (Hz), integration. ^13^C-NMR data were collected at 100 MHz with complete proton decoupling. Chemical shifts were reported in ppm from the tetramethylsilane with the solvent resonance as internal standard. MS spectra were obtained on a Waters Quattro Premier XETM triple quadrupole mass spectrometer and methanol was used to dissolve the sample. Melting points were recorded at SGW X-4 Melting point instrument (Shanghai Precision & Scientific Instrument Co., Ltd, Shanghai, China).

### 3.2. Experimental Procedures

A mixture of isatin **1a** (0.1 mmol), (*E*)-4-phenylbut-3-en-2-one (**2a**, 0.1 mmol), arginine (0.01 mmol) in MeOH (1.0 mL) was stirred for 48 h at 25 °C. After completion of the reaction (TLC), the solvent was removed under vacuum. The crude product was subjected to column chromatography on silica gel using petroleum ether/ethyl acetate = 1:1 as the eluent to give **3a**.

Compounds **3b**–**t** were synthesized by a similar procedure as described for compound **3a**. For the separation of these compounds, the eluent of silica gel column chromatography consisted of appropriate mixtures of petroleum ether and ethyl acetate.

### 3.3. Spectral Data

*(E)-3-Hydroxy-3-(2-oxo-4-phenylbut-3-en-1-yl)indolin-2-one* (**3a**). Yield 98%; White solid; m.p. 159–160 °C; ^1^H-NMR (400 MHz, TMS, DMSO): δ 3.23 (d, *J =* 16.0 Hz, 1H), 3.65 (d, *J =* 16.0 Hz, 1H), 6.05 (s, 1H), 6.74–6.80 (m, 2H), 6.88 (t, *J =* 8.0 Hz, 1H), 7.16 (t, *J =* 8.0 Hz, 1H), 7.28 (d, *J =* 8.0 Hz, 1H), 7.42–7.43 (m, 3H), 7.55 (d, *J =* 16.4 Hz, 1H), 7.67 (m, 2H), 10.24 (s, 1H); ^13^C-NMR (100 MHz, DMSO): δ 47.6, 73.1, 109.4, 121.1, 123.7, 126.4, 128.4, 128.9, 130.5, 131.5, 134.3, 142.5, 142.7, 178.2, 196.2; MS: *m/z* = 316 [M+Na]^+^.

*(E)-3-Hydroxy-3-(4-(4-methoxyphenyl)-2-oxobut-3-en-1-yl)indolin-2-one* (**3b**). Yield 95%; White solid; m.p. 167–168 °C; ^1^H-NMR (400 MHz, TMS, DMSO): δ 3.20 (d, *J =* 16.4 Hz, 1H), 3.63 (d, *J =* 16.4 Hz, 1H), 3.80 (s, 3H), 6.04 (s, 1H), 6.62 (d, *J =* 16.4 Hz, 1H), 6.78 (d, *J =* 7.6 Hz, 1H), 6.88 (t, *J =* 7.6 Hz, 1H), 6.98 (t, *J =* 8.4 Hz, 2H), 7.16 (t, *J =* 7.6 Hz, 1H), 7.27 (d, *J =* 7.2 Hz, 1H), 7.51 (d, *J =* 16.4 Hz, 1H), 7.62 (d, *J =* 8.4 Hz, 2H), 10.23 (s, 1H); ^13^C-NMR (100 MHz, DMSO): δ 47.4, 55.3, 73.1, 109.3, 114.4, 121.1, 123.7, 124.1, 126.8, 128.9, 130.3, 131.5, 142.5, 142.7, 161.2, 178.3, 196.0; MS: *m/z* = 346 [M+Na]^+^.

*(E)-3-(4-(4-Fluorophenyl)-2-oxobut-3-en-1-yl)-3-hydroxyindolin-2-one* (**3c**). Yield 87%; White solid; m.p. 177–178 °C; ^1^H-NMR (400 MHz, TMS, DMSO): δ 3.23 (d, *J =* 16.4 Hz, 1H), 3.66 (d, *J =* 16.4 Hz, 1H), 6.07 (s, 1H), 6.72 (d, *J =* 16.4 Hz, 1H), 6.79 (d, *J =* 7.6 Hz, 1H), 6.88 (t, *J =* 7.6 Hz, 1H), 7.16 (t, *J =* 7.6 Hz, 1H), 7.24–7.29 (m, 3H), 7.56 (d, *J =* 16.4 Hz, 1H), 7.74 (m, 2H), 10.26 (s, 1H); ^13^C-NMR (100 MHz, DMSO): δ = 47.6, 73.1, 109.4, 115.9 (d, *J =* 22 Hz), 121.1, 123.7, 126.3, 128.9, 130.8 (d, *J =* 8 Hz), 130.9 (d, *J =* 3 Hz), 131.5, 141.4, 142.7, 163.3 (d, *J =* 248 Hz), 178.2, 196.2; MS: *m/z* = 334 [M+Na]^＋^.

*(E)-3-(4-(4-Chlorophenyl)-2-oxobut-3-en-1-yl)-3-hydroxyindolin-2-one* (**3d**). Yield 80%; White solid; m.p. 196–197 °C; ^1^H-NMR (400 MHz, TMS, DMSO): δ 3.23 (d, *J =* 16.4 Hz, 1H), 3.65 (d, *J =* 16.4 Hz, 1H), 6.07 (s, 1H), 6.77 (m, 2H), 6.88 (t, *J =* 7.6 Hz, 1H), 7.16 (t, *J =* 7.6 Hz, 1H), 7.27 (d, *J =* 7.2 Hz, 1H), 7.51 (m, 3H), 7.70 (d, *J =*8.4 Hz, 2H), 10.25 (s, 1H);^13^C-NMR (100 MHz, DMSO): δ 47.7, 73.1, 109.4, 121.1, 123.7, 127.0, 129.0, 130.1, 131.4, 133.3, 135.0, 141.1, 142.6, 178.2, 196.2; MS: *m/z* = 350 [M+Na]^＋^.

*(E)-3-(4-(4-Bromophenyl)-2-oxobut-3-en-1-yl)-3-hydroxyindolin-2-one* (**3e**). Yield 82%; White solid; m.p. 205–206 °C; ^1^H-NMR (400 MHz, TMS, DMSO): δ 3.22 (d, *J =* 16.4 Hz, 1H), 3.64 (d, *J =* 16.4 Hz, 1H), 6.05 (s, 1H), 6.79 (m, 2H), 6.88 (t, *J =* 7.6 Hz, 1H), 7.16 (t, *J =* 7.6 Hz, 1H), 7.27 (d, *J =* 7.2 Hz, 1H), 7.52 (d, *J =* 16.0 Hz, 1H), 7.62 (s, 4H), 10.23 (s, 1H); ^13^C-NMR (100 MHz, DMSO): δ = 47.7, 73.1, 109.4, 121.1, 123.7, 123.8, 127.1, 128.9, 130.3, 131.4, 131.9, 133.6, 141.1, 142.6, 178.2, 196.2; MS: *m/z* = 394 [M+Na]^＋^.

*(E)-3-Hydroxy-3-(4-(4-nitrophenyl)-2-oxobut-3-en-1-yl)indolin-2-one* (**3f**). Yield 75%; White solid; m.p. 195–196 °C; ^1^H-NMR (400 MHz, TMS, DMSO): δ 3.25 (d, *J =* 16.4 Hz, 1H), 3.68 (d, *J =* 16.4 Hz, 1H), 6.09 (s, 1H), 6.76 (d, *J =* 7.6 Hz, 1H), 6.89 (t, *J =* 7.6 Hz, 1H), 6.95 (d, *J =* 16.4 Hz, 1H), 7.16 (t, *J =* 7.6 Hz, 1H), 7.28 (d, *J =*7.2 Hz, 1H), 7.63 (d, *J =* 16.4 Hz, 1H), 7.94 (d, *J =*8.8 Hz, 2H), 8.25 (d, *J =* 8.8 Hz, 2H), 10.27 (s, 1H); ^13^C-NMR (100 MHz, DMSO): δ = 47.9, 73.1, 109.4, 121.2, 123.8, 124.0, 129.0, 129.4, 129.9, 131.3 139.7, 140.9, 142.6, 148.0, 178.1, 196.3; MS: *m/z* = 361 [M+Na]^＋^.

*(E)-3-Hydroxy-3-(2-oxo-4-(3-(trifluoromethyl)phenyl)but-3-en-1-yl)indolin-2-one* (**3g**). Yield 86%; White solid; m.p. 114–115 °C; ^1^H-NMR (400 MHz, TMS, DMSO): δ 3.23 (d, *J =* 16.4 Hz, 1H), 3.65 (d, *J =* 16.4 Hz, 1H), 6.06 (s, 1H), 6.78 (d, *J =* 7.6 Hz, 1H), 6.88 (m, 2H), 7.16 (td, *J =* 7.6 Hz, *J =* 1.2 Hz, 1H), 7.27 (d, *J =* 7.6 Hz, 1H), 7.65 (m, 2H), 7.76 (d, *J =*7.6 Hz, 1H), 7.97 (d, *J =*8.0 Hz, 1H), 8.05 (s, 1H), 10.24 (s, 1H); ^13^C-NMR (100 MHz, DMSO): δ 47.9, 73.1, 109.4, 121.1, 123.8, 123.9 (q, *J =* 271 Hz), 124.9 (q, *J =* 14 Hz), 126.6 (q, *J =* 4 Hz), 128.2, 129.0, 129.6, 130.0, 131.4, 132.0, 135.6, 140.5, 142.6, 178.1, 196.2; MS: *m/z* = 384 [M+Na]^＋^.

*(E)-3-(4-(3-Bromophenyl)-2-oxobut-3-en-1-yl)-3-hydroxyindolin-2-one* (**3h**). Yield 85%; White solid; m.p. 173–174 °C; ^1^H-NMR (400 MHz, TMS, DMSO): δ 3.23 (d, *J =* 16.4 Hz, 1H), 3.65 (d, *J =* 16.4 Hz, 1H), 6.08 (s, 1H), 6.78–6.90 (m, 3H), 7.17 (t, *J =* 7.2 Hz, 1H), 7.27 (d, *J =* 6.8 Hz, 1H), 7.37 (t, *J =* 7.6 Hz, 1H), 7.50 (d, *J =*16.4 Hz, 1H), 7.61 (d, *J =* 7.2 Hz, 1H), 7.67 (d, *J =* 7.2 Hz, 1H), 7.91 (s, 1H), 10.26 (s, 1H); ^13^C-NMR (100 MHz, DMSO): δ 47.8, 73.1, 109.4, 121.1, 122.3, 123.7, 127.4, 127.7, 129.0, 130.8, 131.0, 131.4, 132.9, 136.9, 140.7, 142.6, 178.2, 196.2; MS: *m/z* = 394 [M+Na]^＋^.

*(E)-3-(4-(3-Chlorophenyl)-2-oxobut-3-en-1-yl)-3-hydroxyindolin-2-one* (**3i**). Yield 82%; White solid; m.p. 150–151 °C; ^1^H-NMR (400 MHz, TMS, DMSO): δ 3.22 (d, *J =* 16.4 Hz, 1H), 3.65 (d, *J =* 16.4 Hz, 1H), 6.06 (s, 1H), 6.79 (d, *J =* 7.6 Hz, 1H), 6.84 (d, *J =* 16.4 Hz, 1H), 6.89 (d, *J =* 7.6 Hz, 1H), 7.16 (td, *J =* 7.6 Hz, *J =* 0.8 Hz, 1H), 7.27 (d, *J =* 7.2 Hz, 1H), 7.42–7.53 (m, 3H), 7.63 (d, *J =* 7.2 Hz, 1H), 7.77 (s, 1H), 10.24 (s, 1H); ^13^C-NMR (100 MHz, DMSO): δ 47.8, 73.1, 109.4, 121.1, 123.7, 127.0, 127.8, 127.9, 129.0, 130.0, 130.7, 131.4, 133.7, 136.6, 140.7, 142.6, 178.1, 196.2; MS: *m/z* = 350 [M+Na]^＋^.

*(E)-3-Hydroxy-3-(4-(3-methoxyphenyl)-2-oxobut-3-en-1-yl)indolin-2-one* (**3j**). Yield 96%; White solid; m.p. 158–159 °C; ^1^H-NMR (400 MHz, TMS, DMSO): δ 3.21 (d, *J =* 16.0 Hz, 1H), 3.64 (d, *J =* 16.4 Hz, 1H), 3.78 (s, 3H), 6.05 (s, 1H), 6.75–6.79 (m, 2H), 6.88 (t, *J* = 7.6 Hz, 1H), 6.99 (d, *J* = 6.8 Hz, 1H), 7.16 (t, *J =* 7.6 Hz, 1H), 7.23–7.28 (m, 3H), 7.33 (t, *J =* 8.0 Hz, 1H), 7.51 (d, *J =* 16.0 Hz, 1H), 10.23 (s, 1H); ^13^C-NMR (100 MHz, DMSO): δ47.6, 55.2, 73.1, 109.4, 113.1, 116.6, 121.0, 121.1, 123.7, 126.7, 128.9, 129.9, 131.5, 135.7, 142.5, 142.7, 159.6, 178.2, 196.3; MS: *m/z* = 346 [M+Na]^＋^.

*(E)-3-(4-(2-Fluorophenyl)-2-oxobut-3-en-1-yl)-3-hydroxyindolin-2-one* (**3k**). Yield 92%; White solid; m.p. 155–156 °C; ^1^H-NMR (400 MHz, TMS, DMSO): δ 3.24 (d, *J =* 16.4 Hz, 1H), 3.63 (d, *J =* 16.4 Hz, 1H), 6.09 (s, 1H), 6.79 (d, *J =* 7.6 Hz, 1H), 6.84–6.90 (m, 2H), 7.14–7.18 (m, 1H), 7.23–7.30 (m, 3H), 7.45–7.48 (m, 1H), 7.53 (d, *J =* 16.4 Hz, 1H), 7.78 (t, *J =* 7.2 Hz, 1H), 10.27 (s, 1H); ^13^C-NMR (100 MHz, DMSO): δ = 48.1, 73.1, 109.4, 116.1(d, *J* = 22 Hz), 121.2, 121.8, 121.9, 123.8, 125.0 (d, *J* = 3 Hz), 128.4 (d, *J* = 5 Hz), 129.0, 129.2, 131.4, 132.5 (d, *J* = 9 Hz), 133.9 (d, *J* = 3 Hz), 142.6, 160.8 (d, *J* = 250 Hz), 178.2; MS: *m/z* = 334 [M+Na]^＋^.

*(E)-3-(4-(3,4-Dichlorophenyl)-2-oxobut-3-en-1-yl)-3-hydroxyindolin-2-one* (**3l**). Yield 73%; White solid; m.p. 166–167 °C; ^1^H-NMR (400 MHz, TMS, DMSO): δ 3.22 (d, *J =* 16.4 Hz, 1H), 3.63 (d, *J =* 16.4 Hz, 1H), 6.07 (s, 1H), 6.78 (d, *J =* 7.6 Hz, 1H), 6.88 (m, 2H), 7.16 (t, *J =* 7.6 Hz, 1H), 7.27 (d, *J =* 7.6 Hz, 1H), 7.51 (d, *J =* 16.0 Hz, 1H), 7.67–7.69 (m, 2H), 7.99 (s, 1H), 10.25 (s, 1H); ^13^C-NMR (100 MHz, DMSO): δ47.8, 73.0, 109.4, 121.1, 123.7, 128.2, 128.3, 129.0, 130.1, 131.0, 131.4, 131.7, 132.6, 135.3, 139.7, 142.6, 178.1, 196.2; MS: *m/z* = 384 [M+Na]^＋^.

*(E)-3-Hydroxy-3-(4-(naphthalen-2-yl)-2-oxobut-3-en-1-yl)indolin-2-one* (**3m**). Yield 81%; White solid; m.p. 172–173 °C; ^1^H-NMR (400 MHz, TMS, DMSO): δ 3.28 (d, *J =* 16.0 Hz, 1H), 3.71 (d, *J =* 16.4 Hz, 1H), 6.09 (s, 1H), 6.81 (d, *J =* 7.6 Hz, 1H), 6.88–6.93 (m, 2H), 7.17 (t, *J =* 7.6 Hz, 1H), 7.31 (d, *J =* 7.2 Hz, 1H), 7.57 (m, 2H), 7.70 (d, *J =* 16.4 Hz, 1H), 7.82 (d, *J =* 8.4 Hz, 1H), 7.94 (m, 3H), 8.19 (s, 1H), 10.27 (s, 1H); ^13^C-NMR (100 MHz, DMSO): δ 47.8, 73.2, 109.4, 121.1, 123.8, 123.9, 126.6, 126.8, 127.4, 127.7, 128.4, 128.5, 128.9, 130.2, 131.5, 131.9, 132.9, 133.8, 142.4, 142.7, 178.2, 196.2; MS: *m/z* = 344 [M+H]^＋^.

*(E)-3-(4-(Furan-2-yl)-2-oxobut-3-en-1-yl)-3-hydroxyindolin-2-one* (**3n**). Yield 88%; White solid; m.p. 151–152 °C; ^1^H-NMR (400 MHz, TMS, DMSO): δ 3.15 (d, *J =* 16.0 Hz, 1H), 3.55 (d, *J =* 16.0 Hz, 1H), 6.04 (s, 1H), 6.47 (d, *J =* 15.6 Hz, 1H), 6.64 (s, 1H), 6.77 (d, *J =* 7.2 Hz, 1H), 6.88 (m, 1H), 6.95 (m, 1H), 7.16 (m, 1H), 7.25 (m, 1H), 7.33 (d, *J =* 16.0 Hz, 1H), 7.85 (s, 1H), 10.23 (s, 1H); ^13^C-NMR (100 MHz, DMSO): δ 47.7, 73.1, 109.4, 113.0, 116.8, 121.1, 123.1, 123.8, 128.9, 129.0, 131.4, 142.6, 146.1, 150.4, 178.2, 195.6; MS: *m/z* = 306 [M+Na]^＋^.

*(E)-3-Hydroxy-3-(2-oxohept-3-en-1-yl)indolin-2-one* (**3o**). Yield 67%; oil; ^1^H-NMR (400 MHz, TMS, DMSO): δ 0.91 (t, *J =* 4.0 Hz, 3H), 1.40 (m, 2H), 2.16 (m, 2H), 3.12 (d, *J =* 16.0 Hz, 1H), 3.52 (d, *J =* 16.0 Hz, 1H), 5.96–6.04 (m, 2H), 6.77 (d, *J =* 7.6 Hz, 1H), 6.87 (t, *J =* 7.6 Hz, 1H), 7.04 (d, *J =* 16.0 Hz, 1H), 7.15 (t, *J =* 7.6 Hz, 1H), 7.23 (t, *J =* 7.6 Hz, 1H), 10.19 (s, 1H); ^13^C-NMR (100 MHz, DMSO): δ 13.7, 21.9, 30.1, 47.7, 73.1, 109.3, 121.1, 123.6, 128.8, 131.5, 139.1, 142.6, 146.2, 178.2, 196.2; MS: *m/z* = 282 [M+Na]^＋^.

*(E)-1-Benzyl-3-hydroxy-3-(2-oxo-4-phenylbut-3-en-1-yl)indolin-2-one* (**3p**). Yield 99%; White solid; m.p. 57–58 °C; ^1^H-NMR (400 MHz, TMS, DMSO): δ 3.40 (d, *J =* 16.8 Hz, 1H), 3.83 (d, *J =* 16.8 Hz, 1H), 4.85 (d, *J =* 8.0 Hz, 1H), 4.93 (d, *J =* 8.0 Hz, 1H), 6.30 (s, 1H), 6.73–6.81 (m, 2H), 6.95 (t, *J =* 7.2 Hz, 1H), 7.16 (t, *J =* 7.2 Hz, 1H), 7.27 (d, *J =* 7.2 Hz, 1H), 7.33–7.47 (m, 8H), 7.60 (d, *J =* 16.0 Hz, 1H), 7.67 (m, 2H); ^13^C-NMR (100 MHz, DMSO): δ 42.7, 47.7, 72.8, 108.9, 122.0, 123.5, 126.2, 127.2, 128.4, 128.5, 128.9, 129.0, 130.6, 130.9, 134.2, 136.4, 142.9, 143.2, 176.8, 196.3; MS: *m/z* = 384 [M+H]^＋^.

*(E)-3-Hydroxy-5-methyl-3-(2-oxo-4-phenylbut-3-en-1-yl)indolin-2-one* (**3q**). Yield 60%; White solid; m.p. 136–137 °C; ^1^H-NMR (400 MHz, TMS, DMSO): δ 2.19 (s, 3H), 3.23 (d, *J =* 16.4 Hz, 1H), 3.62 (d, *J =* 16.4 Hz, 1H), 6.02 (s, 1H), 6.68 (d, *J =*8.0 Hz, 1H), 6.76 (d, *J =* 16.0 Hz, 1H), 6.96 (d, *J =* 7.6 Hz, 1H), 7.10 (s, 1H), 7.43 (m, 3H), 7.53 (d, *J =* 16.0 Hz, 1H), 7.66 (m, 2H), 10.15 (s, 1H); ^13^C-NMR (100 MHz, DMSO): δ = 20.6, 47.7, 73.2, 109.1, 124.4, 126.4, 128.4, 128.9, 129.1, 129.8, 130.5, 131.5, 134.3, 140.2, 142.5, 178.2, 196.2; MS: *m/z* = 330 [M+Na]^＋^.

*(E)-5-Bromo-3-hydroxy-3-(2-oxo-4-phenylbut-3-en-1-yl)indolin-2-one* (**3r**). Yield 99%; White solid; m.p. 201–202 °C; ^1^H-NMR (400 MHz, TMS, DMSO): δ 3.30 (d, *J =* 16.8 Hz, 1H), 3.75 (d, *J =* 16.8 Hz, 1H), 6.20 (s, 1H), 6.74–6.79 (m, 2H), 7.34–7.59 (m, 6H), 7.67 (m, 2H), 10.40 (s, 1H); ^13^C-NMR (100 MHz, DMSO): δ = 47.4, 73.1, 111.4, 112.9, 126.2, 126.7, 128.5, 128.9, 130.6, 131.5, 134.1, 134.2, 142.1, 142.8, 177.8, 196.3; MS: *m/z* = 394 [M+Na]^＋^.

*(E)-6-Chloro-3-hydroxy-3-(2-oxo-4-phenylbut-3-en-1-yl)indolin-2-one* (**3s**). Yield 93%; Yellow solid; m.p. 188–189 °C; ^1^H-NMR (400 MHz, TMS, DMSO): δ 3.27 (d, *J =* 16.8 Hz, 1H), 3.70 (d, *J =* 16.8 Hz, 1H), 6.15 (s, 1H), 6.75 (d, *J =* 16.0 Hz, 1H), 6.79 (m, 1H), 6.93 (m, 1H), 7.29 (d, *J =* 8.0 Hz, 1H), 7.43 (m, 3H), 7.55 (d, *J =* 16.0 Hz, 1H), 7.67 (m, 2H), 10.41 (s, 1H); ^13^C-NMR (100 MHz, DMSO): δ = 47.4, 72.6, 109.4, 120.8, 125.1, 126.2, 128.5, 128.9, 130.5, 130.6, 133.1, 134.2, 142.8, 144.3, 178.2, 196.3; MS: *m/z* = 350 [M+Na]^＋^.

*(E)-4-Chloro-3-hydroxy-3-(2-oxo-4-phenylbut-3-en-1-yl)indolin-2-one* (**3t**). Yield 92%; Yellow solid; m.p. 193–195 °C; ^1^H-NMR (400 MHz, TMS, DMSO): δ 3.25 (d, *J =* 16.8 Hz, 1H), 4.03 (d, *J =* 16.8 Hz, 1H), 6.22 (s, 1H), 6.76 (m, 2H), 6.86 (d, *J =* 8.4 Hz, 1H), 7.18 (t, *J =* 8.0 Hz, 1H), 7.43 (m, 3H), 7.58 (d, *J =* 16.4 Hz, 1H), 7.68 (m, 2H), 10.50 (s, 1H); ^13^C-NMR (100 MHz, DMSO): δ = 45.8, 73.8, 108.5, 122.0, 125.8, 127.5, 128.5, 128.9, 129.8, 130.6, 134.1, 143.0, 145.0, 177.4, 196.2; MS: *m/z* = 350 [M+Na]^＋^.

## 4. Conclusions

In conclusion, we have developed an efficient approach for the direct preparation of 3-substituted-3-hydroxy-2-oxindoles via an aldol reaction of α,β-unsaturated ketones and isatins using arginine as an organocatalyst. A range of 3-substituted-3-hydroxy-2-oxindoles were obtained in moderate to high yields (up to 99%). The formation of 3-substituted-3-hydroxy-2-oxindole (**3a**) was conﬁrmed by X-ray crystallography. The reaction is simple, the catalyst easily availability, and the procedure convenient with mild reaction conditions, which make it useful. The possible reaction mechanism reveals that the reaction proceeds via a double action process. The 3-substituted-3-hydroxy-2-oxindoles with an additional enone moiety and provide a chance for finding new or improved biological activities and further elaboration of the products. Further study on the antibacterial and antitumor activities of these compounds is underway.
